# Comparison of the Efficacy of EGFR-TKIs Combined with Antiangiogenic Agents between Patients with Exon 19 Deletion and Patients with Exon 21 Leu858 Arg Mutation: A Systematic Review and Meta-Analysis

**DOI:** 10.1155/2022/9399797

**Published:** 2022-10-20

**Authors:** Xin-Bin Pan, Fa-Song Liang, Qin-Yu Tang, Huan-Wei Liang, Xiao-Dong Zhu

**Affiliations:** Department of Radiation Oncology, Guangxi Medical University Cancer Hospital, Nanning, Guangxi 530021, China

## Abstract

**Purpose:**

To compare the efficacy of EGFR-TKIs combined with antiangiogenic agents between non-small cell lung cancer patients with exon 19 deletion and patients with exon 21 Leu858 Arg mutation.

**Methods:**

Electronic databases (PubMed, Embase, and the Cochrane Central Register of Controlled Trials) were systematically searched for studies published until March 2022. Randomized control trials comparing the survival of EGFR-TKIs plus antiangiogenic agents with EGFR-TKI were extracted. The primary endpoint was progression-free survival (PFS).

**Results:**

Five randomized control trials involving 1533 patients were as follows: 818 patients had exon 19 deletion, and 715 patients with exon 21 Leu858 Arg mutation. The methodological quality of the 5 randomized control trials was high. EGFR-TKIs plus antiangiogenic agents improved PFS in patients with exon 19 deletion (hazard ratio [HR] = 0.62, 95% confidence interval [CI]: 0.51–0.75) and exon 21 Leu858 Arg mutation (HR = 0.61, 95% CI: 0.50–0.75). PFS did not differ between the exon 19 deletion and exon 21 Leu858 Arg mutation groups (*Z* = 0.07, *P*=0.94).

**Conclusions:**

PFS was comparable between patients receiving EGFR-TKIs combined with antiangiogenic agents with exon 19 deletion and those with exon 21 Leu858 Arg mutation.

## 1. Introduction

Lung cancer is a leading cause of death worldwide, accounting for 18.0% of total cancer-related deaths [1]. Approximately 85% of cases of lung cancer are non-small cell lung cancer (NSCLC). Among NSCLC patients, 60% have metastatic disease at the time of diagnosis [2]. Epidermal growth factor receptor (EGFR) mutation-driven NSCLC occurs in 10–20% of white patients and 40–60% of Asian patients [3, 4].

Although several trials established EGFR tyrosine kinase inhibitor (TKI) therapy as standard treatment for EGFR-positive NSCLC patients [5–8], the median progression-free survival (PFS) was approximately 1 year as a result of acquired TKI therapeutic resistance [5–10]. To improve PFS, TKIs combined with antiangiogenic agents have been investigated. Several trials have suggested that the addition of antiangiogenic agents to TKIs significantly reduces the risk of disease progression [11–21].

However, it is unclear whether the efficacy of TKIs combined with antiangiogenic agents is similar between patients with exon 19 deletion and patients with exon 21 Leu858 Arg mutation. Several trials have reported that patients with exon 21 Leu858 Arg mutation are more likely to benefit from TKI combined with antiangiogenic agents than patients with exon 19 deletion [17, 19, 21]. In contrast, other studies have suggested that patients with exon 19 deletion are more likely to benefit from TKI combined with antiangiogenic agents than patients with exon 21 Leu858 Arg mutation [16, 18, 20]. Thus, the current systematic review was conducted to compare the PFS between NSCLC patients treated with TKI combined with antiangiogenic agents who had exon 19 deletion and those with exon 21 Leu858 Arg mutation.

## 2. Materials and Methods

### 2.1. Data Sources and Searches

The PubMed, Embase, and Cochrane Central Register of Controlled Trials were systematically searched up to March 2022. The search was performed in accordance with the Preferred Reporting Items for Systematic Reviews and Meta-analyses (PRISMA) reporting guidelines [22, 23]. The main search terms and their combinations included NSCLC, non-small cell lung cancer, EGFR, epidermal growth factor receptor, TKI, a tyrosine kinase inhibitor, antiangiogenic agents, VEGF, and VEGFR. Abstracts from the American Society of Clinical Oncology (ASCO), European Society of Medical Oncology (ESMO), and International Association of Lung Cancer websites were also reviewed. Two researchers (PXB and LFS) independently carried out the literature retrieval. If multiple articles covered the same study population, the study with the most recent and complete survival data was utilized. Any disagreements between the two reviewers were resolved by consulting a third reviewer (ZXD).

### 2.2. Study Selection

Studies were included if they met the following criteria: (1) randomized clinical trials; (2) untreated patients with cytologically or histologically confirmed advanced EGFR-mutant NSCLC; (3) compared EGFR-TKIs plus antiangiogenic agents with EGFR-TKI monotherapy in the first-line setting; and (4) reported PFS of patients with exon 19 deletion and exon 21 Leu858 Arg mutation. Studies failing to meet these criteria were excluded.

### 2.3. Data Extraction and Quality Assessment

Data extraction was performed by 2 authors (LFS and TQY). Two authors (PXB and LHW) separately assessed the methodological quality of the included studies. The methodological quality of randomized clinical trials was evaluated by the Cochrane Risk of Bias tool [24], which assesses the following seven domains: (1) random sequence generation; (2) allocation concealment; (3) blinding of participants and personnel; (4) blinding of outcome assessment; (5) incomplete outcome data; (6) selective reporting; and (7) other bias. All disagreements were resolved by discussion and consensus.

### 2.4. Statistical Analysis

The pooled hazard ratios (HRs) for PFS and its 95% confidence intervals (CIs) were used to measure the treatment outcome. The *I*^2^-statistic was used to determine the level of statistical heterogeneity between studies. If there was no statistical heterogeneity (*I*^2^ < 50%, *P* ≥ 0.1) among studies, a fixed effects model was used for HR analysis. If there was statistical heterogeneity (*I*^2^ ≥ 50%, *P* < 0.1) among studies, a random effects model was used. Forest plots were generated to show the estimated HRs, representing the theoretical gain in absolute percentage on the basis of PFS. The upper limit and lower limit of 95% CIs were calculated. The stability of the results was estimated using a sensitivity analysis by the exclusion of a particular trial from the analysis. Publication bias was assessed by the funnel plot and Begg's and Egger's tests.

All statistical analyses were performed using SPSS Statistics Version 26.0 software (IBM Co., Armonk, NY, USA) and *R* software version 4.1.3 (https://www.R-project.org). *P* values were two-tailed. Values of *P* < 0.05 were considered statistically significant.

## 3. Results

### 3.1. Characteristics of the Included Trials

**Figure 1:**
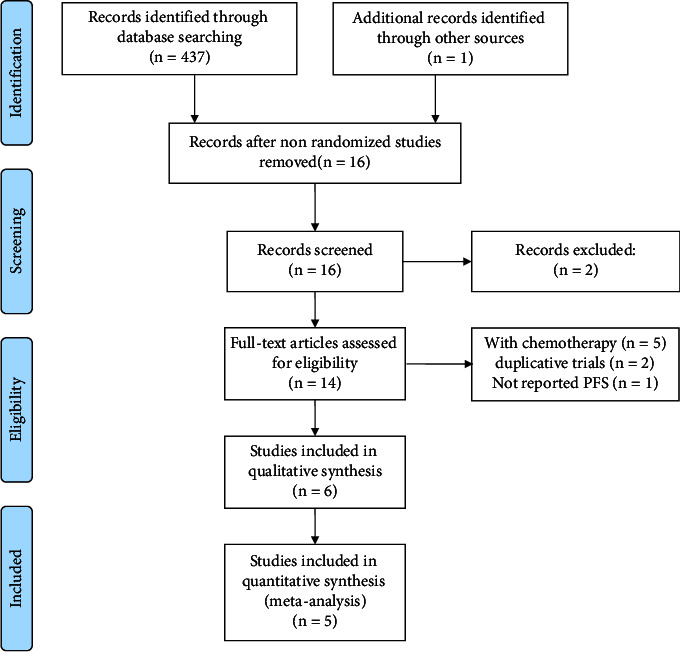
Flowchart depicting study selection.

Figure 1 shows the process of study selection. This study screened 438 studies according to the primary search strategy. Six trials were included in this systematic review [16–21]. Five studies were included in the meta-analysis [16–19, 21].

**Figure 2:**
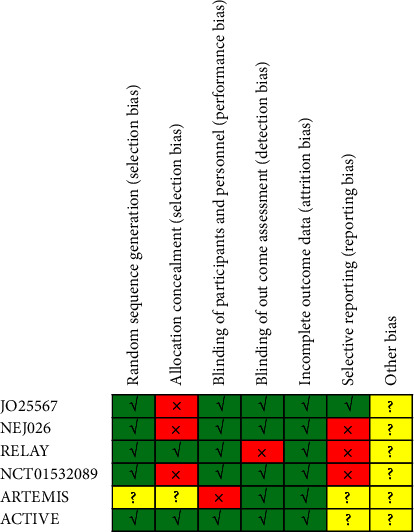
Risk of bias assessment of included studies.

A total of 1533 patients were included 818 patients had exon 19 deletion, and 715 patients had exon 21 Leu858 Arg mutation.  Table 1 summarizes the characteristics of the 6 included studies.  Table 2 lists the primary endpoint of the 6 trials. Two studies reported overall survival (OS) [18, 20].  Figure 2 shows the methodological quality of the 6 included studies. Among the 6 studies, 2 trials were phase 2 randomized clinical trials, and 4 studies were phase 3 randomized clinical trials.

### 3.2. PFS of Patients with Exon 19 Deletion

**Figure 3:**
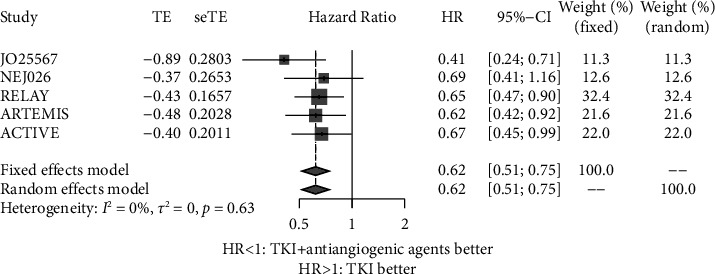
Forest plot of the hazard ratio of the progression-free survival of exon 19 deletion receiving EGFR-TKIs plus antiangiogenic agents and EGFR-TKIs. HR: hazard ratio; CI: confidence interval.

PFS data of patients with exon 19 deletion were available from 5 trials [16–19, 21]. There was no significant heterogeneity among the 5 trials (*P*=0.63, *I*^2^ = 0.00%); therefore, the fixed effects model was used for meta-analysis. As shown in Figure 3, patients with exon 19 deletion receiving EGFR-TKIs plus antiangiogenic agents had longer PFS than patients receiving EGFR-TKI monotherapy (HR = 0.62, 95% CI: 0.51–0.75).

**Figure 4:**
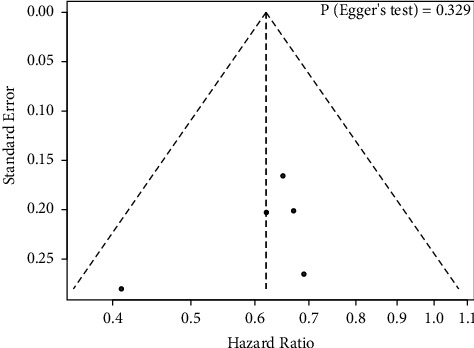
Publication bias assessment of exon 19 deletion receiving EGFR-TKIs plus antiangiogenic agents and EGFR-TKIs.

**Figure 5:**
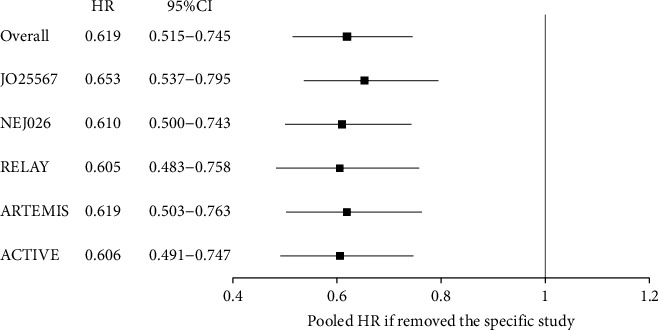
Sensitivity analysis of exon 19 deletion receiving EGFR-TKIs plus antiangiogenic agents and EGFR-TKIs.

There was no evidence of apparent publication bias according to Egger's test (*P*=0.329) (Figure 4). Furthermore, sensitivity analysis was conducted by removing one study at a time from the analysis, and the results indicated that the conclusions were robust (Figure 5).

### 3.3. PFS of Patients with Exon 21 Leu858 Arg Mutation

**Figure 6:**
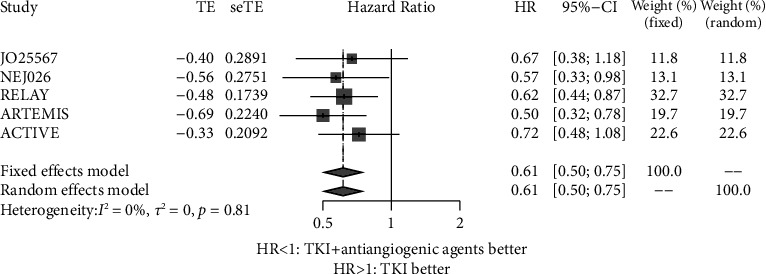
Forest plot of the hazard ratio of the progression-free survival of exon 21 Leu858 Arg mutation receiving EGFR-TKIs plus antiangiogenic agents and EGFR-TKIs. HR: hazard ratio; CI: confidence interval.

PFS data of patients with exon 21 Leu858 Arg mutation were available from 5 trials [16–19, 21]. No significant heterogeneity among the 5 trials was found (*P*=0.81, *I*^2^ = 0.00%). Thus, the fixed effects model was used for meta-analysis.  Figure 6 shows that patients with exon 21 Leu858 Arg mutation who received EGFR-TKIs plus antiangiogenic agents had longer PFS than patients who received EGFR-TKI monotherapy (HR = 0.61, 95% CI: 0.50–0.75).

**Figure 7:**
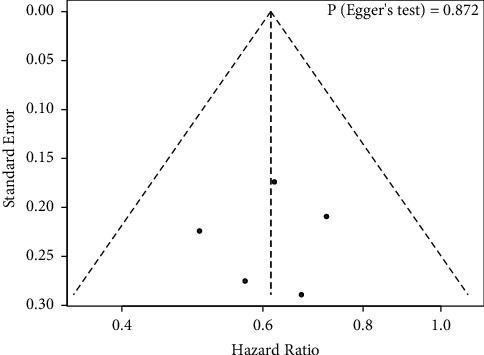
Publication bias assessment of exon 21 Leu858 Arg mutation receiving EGFR-TKIs plus antiangiogenic agents and EGFR-TKIs.

**Figure 8:**
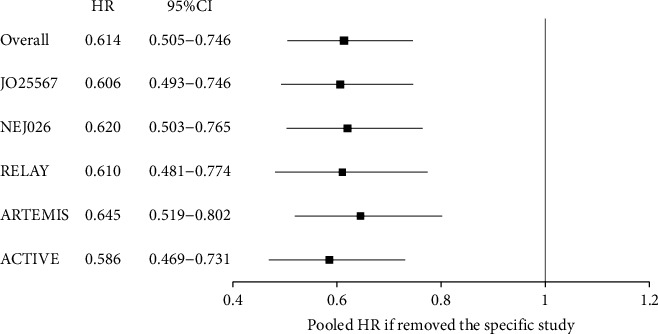
Sensitivity analysis of exon 21 Leu858 Arg mutation receiving EGFR-TKIs plus antiangiogenic agents and EGFR-TKIs.

There was no evidence of apparent publication bias according to Egger's test (*P*=0.872) (Figure 7). Furthermore, sensitivity analysis was conducted by removing one study at a time from the analysis, and the results indicated that the conclusions were robust (Figure 8).

### 3.4. Comparison of PFS among Patients Receiving EGFR-TKIs plus Antiangiogenic Agents with the Exon 19 Deletion and Those with Exon 21 Leu858 Arg Mutation

The *Z*-test was used to compare the PFS between patients receiving EGFR-TKIs plus antiangiogenic agents with exon 19 deletion and those with exon 21 Leu858 Arg mutation. The null hypothesis was that the PFS was comparable between patients with exon 19 deletion and those with exon 21 Leu858 Arg mutation receiving EGFR-TKIs plus antiangiogenic agents. A two-tailed*P* value less than 0.05 was considered to indicate statistical significance.

The *Z* value was 0.07 (*P*=0.94). The results suggested that patients with exon 19 deletion and patients with exon 21 Leu858 Arg mutation receiving EGFR-TKIs plus antiangiogenic agents had a comparable PFS. Similarly, the NCT01532089 trial revealed the same result. [20] Although EGFR-TKIs plus antiangiogenic agents improved the PFS of patients with exon 19 deletion compared to patients with exon 21 Leu858 Arg mutation, the difference between the two groups was not statistically significant (HR = 0.83, 95% CI: 0.47–1.47; *P*=0.53).

### 3.5. Comparison of OS between Exon 19 Deletion and Exon 21 Leu858 Arg Mutation

OS data of patients with exon 19 deletion were available from 2 trials [20, 25]. The NCT01532089 trial reported that the OS of patients with exon 19 deletion receiving EGFR-TKIs plus antiangiogenic agents was better than that of patients with exon 21 Leu858 Arg mutation (HR = 0.34, 95% CI: 0.16–0.72) [20]. In contrast, JO25567 suggested that no differences were observed between patients receiving EGFR-TKIs and those receiving EGFR-TKIs plus antiangiogenic agents [25]. The HRs were 0.79 (95% CI: 0.44–1.44) and 0.83 (95% CI: 0.46–1.49) in the exon 19 deletion and exon 21 Leu858 Arg mutation groups, respectively.

## 4. Discussion

TKIs have been proven to be an effective first-line treatment for patients with EGFR mutation-driven NSCLC. However, the efficacy of TKIs varies among individual patients. Several randomized controlled phase 3 studies revealed that TKIs were more effective in patients harboring exon 19 deletion than in patients harboring exon 21 Leu858 Arg mutation [7, 9, 26–28]. These findings suggested that patients with exon 19 deletion were more sensitive to TKI treatment than those with exon 21 Leu858 Arg mutation. It was suggested that patients with exon 21 Leu858 Arg mutation needed more intense treatment to achieve a similar prognosis to patients with exon 19 deletion.

A possible explanation for the worse prognosis of the exon 21 Leu858 Arg mutation may be that this mutation exhibited a higher proportion of comutations than the exon 19 deletion [29]. The BENEFIT study reported that patients with EGFR co-mutations had a worse prognosis than those with EGFR mutations alone [30]. Another possible explanation may be that patients with exon 21 Leu858 Arg mutation were more likely to have T790 M mutations than patients with exon 19 deletion [31]. NSCLC patients with T790 M mutations before systemic treatment had worse PFS when treated with first-generation TKIs [32].

The FLAURA trial demonstrated that the third-generation TKI osimertinib shows superior efficacy compared to standard EGFR-TKIs in the first-line treatment of EGFR mutation-positive advanced NSCLC [28]. It was also reported that the HR of PFS was 0.43 (95% CI: 0.32–0.56) and 0.51 (95% CI: 0.36–0.71) in the exon 19 deletion and exon 21 Leu858 Arg mutation groups, respectively. The results also suggested that patients with exon 21 Leu858 Arg mutation might have worse PFS than those with exon 19 deletion receiving third-generation TKIs.

Whether osimertinib plus antiangiogenic agents could further improve the PFS of patients with exon 21 Leu858 Arg mutation remains unclear. A phase 1 study comparing the efficacy of ramucirumab plus osimertinib reported that the objective response rate was 76%, and the median PFS was 11.0 months (90% CI: 5.5–19.3) [33]. However, the WJOG-8715 L trial compared osimertinib plus bevacizumab vs. osimertinib alone, and the combination treatment did not lead to prolonger PFS in patients with advanced lung adenocarcinoma with EGFR T790 M mutation (HR = 1.44, 95% CI: 0.83–2.52; *P*=0.20). [34] Similarly, the BOOSTER trial also revealed that osimertinib plus bevacizumab did not improve the median PFS (HR = 0.96, 95% CI: 0.68–1.37) or OS (HR = 1.03, 95% CI: 0.67–1.56) compared to osimertinib alone [35]. These results suggested that the third-generation TKI plus antiangiogenic agents did not improve survival in patients with EGFR mutation-driven NSCLC.

However, the WJOG-8715 L and BOOSTER trials did not report the efficacy of osimertinib combined with antiangiogenic agents in patients with exon 19 deletion and exon 21 Leu858 Arg mutation [34, 35]. On the other hand, the two trials enrolled patients with EGFR mutation-driven NSCLC that acquired T790 M mutations after failure on previous EGFR-TKI therapy. Thus, our meta-analysis could not extract sufficient data to perform subgroup analysis.

Our meta-analysis suggested that combining antiangiogenic agents with TKIs improved PFS in NSCLC patients with exon 19 deletion and patients with exon 21 Leu858 Arg mutation. Moreover, PFS was not different between the two subgroups. The current meta-analysis revealed that both patients with exon 19 deletion and patients with exon 21 Leu858 Arg mutation could benefit from EGFR-TKIs plus antiangiogenic agents. Thus, NSCLC patients with exon 21 Leu858 Arg mutation were recommended to receive EGFR-TKIs plus antiangiogenic agents.

It was reported that patients with exon 19 deletion had a better OS than those with exon 21 Leu858 Arg mutation. [36] The NCT01532089 trial revealed a similar result: the OS of patients with exon 19 deletion receiving EGFR-TKIs plus antiangiogenic agents was better than that of patients with the 21 Leu858 Arg mutation (HR = 0.34, 95% CI: 0.16–0.72) [20]. In contrast, the HRs were comparable between the two subgroups (0.79 vs. 0.83). We did not have a sufficient amount of data to draw conclusions regarding OS; therefore, whether OS was comparable between the two subgroups receiving EGFR-TKIs plus antiangiogenic agents remains unclear. More trials are needed to verify the results.

The advantage of our meta-analysis was that the included studies were all randomized clinical trials with high quality. No evidence of apparent publication bias was observed. The sensitivity analysis indicated stable results by the exclusion of a particular trial from the analysis. However, limitations should be considered. Our meta-analysis was based on the PFS of patients with exon 19 deletion and exon 21 Leu858 Arg mutation reported from the subgroup analysis. The essence of subgroup analysis is exploratory. The results need to be verified in future randomized clinical trials.

In conclusion, PFS was comparable between patients with exon 19 deletion and exon 21 Leu858 Arg mutation receiving EGFR-TKIs combined with antiangiogenic agents.

## Figures and Tables

**Table 1:** Baseline characteristics of included studies.

Trials	Authors	Source (year)	Region	Study type	Study population	Treatments	Sample size		Primary endpoint
							Exon 19 deletion	Exon 21 Leu858 Arg mutation	
JO25567	Seto et al.	Lancet Oncol	Japan	Phase 2 RCT	IIIB/IV or recurrent	Erlotinib 150 mg/d	40	37	PFS
		2014				Erlotinib 150 mg/d + bevacizumba 15 mg/kg Q3w	40	35	
NEJ026	Saito et al.	Lancet Oncol	Japan	Phase 3 RCT	IIIB/IV or recurrent	Erlotinib 150 mg/d	55	57	PFS
		2019				Erlotinib 150 mg/d + bevacizumba 15 mg/kg Q3w	56	56	
RELAY	Nakagawa et al.	Lancet Oncol	Worldwide	Phase 3 RCT	IV or recurrent	Erlotinib 150 mg/d	120	105	PFS
		2019				Erlotinib 150 mg/d + ramucirumab 10 mg/kg Q2w	123	99	
NCT01532089	Stinchcombe et al.	JAMA Oncol	USA	Phase 2 RCT	IV	Erlotinib 150 mg/d	30	15	PFS
		2019				Erlotinib 150 mg/d + bevacizumba 15 mg/kg Q3w	29	14	
ARTEMIS	Zhou et al.	Cancer cell	China	Phase 3 RCT	IIIB/IV or recurrent	Erlotinib 150 mg/d	79	75	PFS
		2021				Erlotinib 150 mg/d + bevacizumba 15 mg/kg Q3w	82	75	
ACTIVE	Zhao et al.	J thorac Oncol	China	Phase 3 RCT	IIIB/IV	Gefitinib 250 mg/d	83	73	PFS
		2021				Gefitinib 250 mg/d + apatinib 500 mg/d	81	74	

RCT: randomized controlled trial. PFS: progression-free survival.

**Table 2:** Survivals of patients with exon 19 deletion and exon 21 Leu858 Arg mutation receiving EGFR-TKIs plus antiangiogenic agents and EGFR-TKIs.

Trials	EGFR mutation type	PFS		OS	
		HR	95% CI	HR	95% CI
JO25567	Exon 19 deletion	0.41	0.24–0.72	0.79	0.44–1.44
	Exon 21 Leu858 arg mutation	0.67	0.38–1.18	0.83	0.46–1.49
NEJ026	Exon 19 deletion	0.69	0.41–1.16		
	Exon 21 Leu858 arg mutation	0.57	0.33–0.97		
RELAY	Exon 19 deletion	0.65	0.47–0.90		
	Exon 21 Leu858 arg mutation	0.62	0.44–0.87		
NCT01532089	Exon 19 deletion	0.83	0.47–1.47	0.34	0.16–0.72
	Exon 21 Leu858 arg mutation	Reference		Reference	
ARTEMIS	Exon 19 deletion	0.62	0.42–0.93		
	Exon 21 Leu858 arg mutation	0.50	0.32–0.77		
ACTIVE	Exon 19 deletion	0.67	0.45–0.99		
	Exon 21 Leu858 arg mutation	0.72	0.48–1.09		

EGFR: epidermal growth factor receptor. HR: hazard ratio. CI: confidence interval. PFS: progression-free survival. OS: overall survival.

## Data Availability

The data that support the findings of this study are available from the corresponding author upon reasonable request.
